# Nurse-led renal cancer follow-up is safe and associated with high patient satisfaction—an audit from the East of England

**DOI:** 10.3332/ecancer.2019.955

**Published:** 2019-07-30

**Authors:** Amy Sibbons, Rajiv Pillai, John Corr, Satyendra Persaud

**Affiliations:** 1 Department of Urology, Colchester General Hospital, Colchester, Essex, CO4 5JL, UK; 2 Division of Clinical Surgical Sciences, University of the West Indies, Trinidad and Tobago

**Keywords:** nurse-led, renal cancer, follow up

## Abstract

**Background:**

With more people diagnosed and dying from renal cancers in England than ever before, treatment and follow-up post-surgery is of paramount importance. We have instituted a nurse-led follow-up service for renal cancers as a way to improve efficiency and make better use of clinic time. This is our first attempt to audit our service.

**Objectives:**

One of the main objectives of this project was to measure compliance of a nurse-led renal surveillance clinic against an established institutional follow-up protocol which was based on current European Association of Urology guidelines. We also aimed to assess patient satisfaction with nurse-led care.

**Patients and Methods:**

A total of 89 patients with low/intermediate-risk kidney cancers who were on the nurse-led renal surveillance database following nephrectomy or partial nephrectomy were placed on a database. This was then audited for adherence to the clinic protocol. These same patients were subsequently sent patient satisfaction questionnaires.

**Results:**

The audit revealed high levels of compliance against the renal clinic protocol as well as positive feedback from the patient satisfaction questionnaire. Ninety-five percent said they felt either at ease or very at ease speaking to the nurse specialist. No one was dissatisfied with their consultations with 86% being very satisfied and 14% fairly satisfied. This was reinforced further by 100% of patients feeling that they could discuss all aspects of their condition with the Uro-oncology Clinical Nurse Specialist (UOCNS). Ninety-seven percent felt that they had adequate time with the nurse.

**Conclusion:**

Nurse-led follow-up, in our setting, was noted to be safe and effective and was associated with high levels of patient satisfaction. This study adds to the growing body of work on the efficacy of nurse-led care.

## Introduction

Over 12,000 new cases of kidney cancer occur in the UK alone each year and 85% of these are renal cell cancers. A variety of treatment options are available for someone diagnosed with a renal cancer but surgery offers the only real hope for a cure and consists of either a partial or radical nephrectomy [2]. At least six in ten patients, if deemed fit enough, choose one of these options [1]. In contemporary series, 10-year survival for localised renal cancer is well over 90% [3]. Intensive surveillance of this cohort of patients for disease recurrence is, therefore, of paramount importance. However, with the continued increase in cancer survivors and the ongoing associated financial strain on resources, issues with capacity continue to increase. As Helgesen [4] related, specialist nurse-led follow-up is more cost-effective and both safe and of a very high quality. With this in mind, the role of the clinical nurse specialist is utilised to have a greater therapeutic effect in clinics as they take the load off the doctors, thereby increasing their capacity to see newly diagnosed patients [5].

In order to reduce the consultants’ workload and ensure effective, holistic care for patients post treatment, the Post-operative Renal Surveillance Clinic was created in 2014. The clinic sees patients for follow-up appointments after either radical or partial nephrectomy surgery for histologically proven renal cell carcinomas at either 3, 6 or 12 monthly intervals depending upon their stage, grade and original diagnosis. The clinic is run by two clinical nurse specialists on a weekly basis and consists on average of six patients per clinic, utilising 30-minute slots.

All referrals are made by a consultant/associate specialist/senior registrar in writing by letter or via local multi-disciplinary team (MDT) /special MDT outcome plan. The patient must be informed of the plan to see a specialist nurse. Referrals will be screened by the Uro-oncology Clinical Nurse Specialist (UOCNS) to ensure they comply with the predetermined inclusion and exclusion criteria (Appendix). Any referrals that do not comply with inclusion criteria will be returned to original source of referral. When a referral is deemed suitable by the UOCNS, an appointment will be booked by the UOCNS secretary, UOCNS or outpatient department reception staff.

The UOCNS adhere to the clinic follow-up protocol with is based on European Association of Urology guidelines. This nurse-led clinic facilitates integration of care between specialist nurses and doctors, with available access to consultants as appropriate. This allows the release of time for the consultants to meet clinical demand and provide medical expertise in other areas where patients may be more complex or require complicated diagnostics.

We feel that our programme has been a success and may be a model which can be reproduced elsewhere. This work represents our first attempt to objectively audit the clinic.

## Aims and objectives

The main aim of the audit is to measure compliance and quality of patient care against the renal clinic protocol. The key elements of the audit criteria sampled were as follows:

- appropriateness and timing of imaging investigation
- adherence to the timing of follow-up visits
- documentation of the reasons, if any, for any deviations from the prescribed schedule

The second part of the project adopted the form of qualitative primary research with a structured questionnaire measuring the satisfaction of patients who attend the clinic.

## Study method

For the audit, the records of all the patients enrolled in the clinic since its inception were reviewed. Patient care was compared to our pre-set clinic protocol which is based on current European Association of Urology guidelines. Data were collected on patient demographics and compliance with respect to appropriate follow-up, imaging and blood investigations. Patients undergoing nephroureterectomy for urothelial carcinoma were excluded from this audit as they follow a different protocol.

A separate questionnaire was created to capture patient satisfaction with and attitudes towards nurse-led care. This questionnaire was piloted among six members of a local support group at their committee meeting. The feedback was reviewed and changes were then made before the questionnaires were posted to all of the patients who are on the renal surveillance database. The patient satisfaction questionnaires were sent out to everyone on the database currently enrolled in the clinic. To minimise recall bias, patients who had been discharged from the clinic were not included in this section of the audit.

## Results

### Audit

A total of 89 patients were audited from the renal surveillance database. The ages of patients ranged from 41 years being the youngest to 90 years being the oldest. The average age of the patients audited was 67 years and 1 month with a standard deviation of 10.78. There were 46 partial nephrectomies and 44 radical nephrectomies with one patient undergoing both procedures. In total, 177 clinic appointments have been recorded as being conducted from the initiation of the clinic in 2014 until the start of the audit in May 2017.

### Radiology scans

Of the 89 patients audited, 87 (97.8%) had the correct scan requested as per the European Association of Urology (EAU) guidance, prior to their clinic appointment. The remaining two went on to develop metastasis, and were hence discussed by the MDT and recommended to have further scans which did not form part of the routine protocol. Of these 89 patients, a total of 82 (92.1%) patients had their scan requested at the correct time interval post-surgery according to the EAU protocol. Of the seven patients breaching protocol, three patients were scanned early due to the development of metastasis and three patients developed post-operative seromas or fluid collections and were hence recommended for closer follow-up, with scans being done outside of protocol.

### Blood investigations

Eighty-seven patients of 89 (97.8%) had the correct blood tests requested according to the clinic protocol. These should include tests to monitor the patient’s haemoglobin, creatinine and estimated glomerular filtration rate. The two that did not were referred to oncology, due to metastases, before their bloods were due.

### Follow-up requested at the correct time according to the protocol

A total of 83 patients (93.3%) had the correct follow-up according to the EAU protocol of either 6 or 12 months depending on the patient. Of the remaining six who didn’t, two were the patients that developed metastasis and were seen sooner than the protocol recommends and then referred on. One had lung nodules which were kept on a more intense surveillance programme of 6 monthly checks but remained under the care of the UOCNS and the remaining three developed seromas and so were also seen sooner.

### Patient satisfaction survey

Thirty-five out of a total of 68 patients returned their questionnaires—a 51% return rate. Eighty-six percent of patients were aware of the UOCNS role and 97% of patients said that the UOCNS introduced herself to them. One-hundred percent of patients were willing to see the UOCNS, with 97% of these patients saying they had been seen in a timely way**.** Ninety-five percent said they felt either at ease or very at ease speaking to her.

Only 53% preferred seeing the nurse over the doctor with 39% unsure 6% indicating they would prefer the doctor. Our questionnaire had additional spacing to encourage patients to elaborate on their answers. Comments with regards to this question ranged from one patient saying ‘*Not sure - feel a doctor would make decisions and the nurse makes notes*’. To other more favourable ones stating ‘*Yes, because Doctors don't always seem to have the time—I felt at ease with the nurse and felt a bit guilty as I was spending so much time with her*’ and another preferring seeing the nurse because ‘*Yes—if only because I was seen on time not two hours late’.*

No one was dissatisfied with their consultations with 86% being very satisfied and 14% fairly satisfied. This was reinforced further by 100% of patients feeling that they could discuss all the aspects of their condition with the UOCNS and 97% feeling that they had adequate time with the nurse—*‘I was not rushed and the clinical nurse specialist put me at ease and answered all of my questions well’*.

The clinic surroundings were found to be comfortable with 94% of patients agreeing and 6% dissatisfied with the setting. Only 6% of patients experienced problems with scan appointments, the other 94% were satisfied. Of the patients who were dissatisfied the reasons were to do with radiology bookings rather than the clinic itself.

Overall, 97% of respondents indicated that the nurse met their needs for follow-up.

## Discussion

Overall the results were positive. The audit revealed that the UOCNS met the criteria of the protocol more often than not and followed it accurately, using clinical judgement when required to refer to other specialities and ask for assistance when needed. In the few instances of a breach in protocol, there were clear reasons for doing so.

While we are aware of other centres offering nurse-led follow-up for renal cancer, there is a paucity of published data in this area. However, the concept of nurse-led care and follow-up has been well applied to prostate cancer [6–9]. In fact, we have previously described our experience with nurse-led active surveillance for prostate cancer [10]. We hope that this study could serve as an impetus for the extension of this concept to renal cancers.

The notion that nurse-led care is an efficient use of outpatient time has been described by other authors. Faithful [11] noted that nurse-led care is efficient and ultimately decreases waiting times. Nurse-led care also frees up time in consultant clinics and allows them to spend time with complicated patients [7, 8].

Additionally, we believe that our nurse-led clinic provides continuity for patients as it establishes contact with a dedicated cancer nurse specialist who will then be a point of contact for that patient throughout his care. This also facilitates the building of rapport between patient and care giver and our high rates of patient satisfaction are perhaps reflective of this. Consistency is valued by patients and Jones [12] found that 93% of patients related that this was an important issue to them. Lewis *et al* [11] and Faithful *et al* [13] found that men formed trusting relationships with their practitioners and these men had had high levels of satisfaction. Patients also expressed confidence in our nurse-led service and this has also been noted among patients in other nurse-led cancer services [6, 9, 14].

In terms of advancements of the clinic in the future, we did ask whether patients would prefer telephone follow-up over a clinic appointment and this was not met with much enthusiasm—only 33% said yes with an overwhelming 56% saying no. The comments made with regards to this consisted of a mixture of practical ones, for example patients being hearing impaired so not being able to hear on the phone and patients living nearby so travelling to clinic is not a problem. Other patients felt a cancer follow-up was too serious to do over the phone or that they preferred to experience all aspects of communication not just verbal as they felt face to face more reassuring. This is supported in a study by Helgesen *et al* [4] who demonstrated that patients favour nurse-led follow-up as it reinforces continuity of care but do not prefer a telephone-led follow-up as they found it unreliable and not appropriate. Interestingly we have also provided a telephone follow-up service for prostate cancer and this has been met with high levels of patient satisfaction.

One limitation of our work was poor participation in the patient satisfaction section of the audit—just over 50%. There is also the potential for bias when one is surveying patients one still cares for, despite the survey being anonymous. We also recognise that satisfaction is relative and is very subjective and that no tool exists to capture it with any reliability.

## Conclusion

Nurse-led follow-up, in our setting, was noted to be safe and effective and was associated with high levels of patient satisfaction. This study adds to the growing body of work on the efficacy of nurse-led care.

## Conflicts of interest

The authors have no conflicts of interest to declare.

## Funding statement

The authors received no funding or compensation for this project.

## Appendix

### Inclusion criteria

The acceptance criteria for the clinic include all the patients who have had a partial nephrectomy or open/laparoscopic radical nephrectomy with histologically proven Fuhrman grade. Criteria for referral to the clinic are outlined below:

- **Fuhrman G1-4, pT1 RCC low/intermediate risk)** who have attended a 6 week post-operative appointment with the urology consultant and have been made aware of their histological diagnosis.
- **Fuhrman G1-4, pT2 RCC (intermediate risk)** and have attended a 6 week post-operative appointment with the urology consultant/ AS/ SpR and have been made aware of their histological diagnosis.
- **Furhman G1, pT3 RCC (intermediate risk)** and have attended a 6 week and 6 month post-operative appointment with the urology consultant/ AS/ SpR and have been made aware of their histological diagnosis.

### Exclusion criteria

- Patients with Fuhrman G 2-4 pT3/ T4 RCC (high risk)
- Patients with node positive disease
- Patient with metastatic disease
- Patients who have complex medical co-morbidities that require active medical management

## References

[1] UK CR (2015). Cancer Research UK.

[2] Escudier B, Porta C, Schmidinger M (2019). Renal cell carcinoma: ESMO clinical practice guidelines for diagnosis, treatment and follow-up. Ann Oncol.

[3] Gettman MT, Blute ML, Spotts B (2001). Pathologic staging of renal cell carcinoma: significance of tumor classification with the 1997 TNM staging system. Cancer.

[4] Helgesen F, Andersson SO, Gustafsson O (2000). Follow-up of prostate cancer patients by on-demand contacts with a specialist nurse a randomized study. Scand J Urol Nephrol.

[5] Willard C, Luker K (2007). Working with the team: strategies employed by hospital cancer nurse specialists to implement their role. J Clin Nurs.

[6] Wade J, Holding PN, Bonnington S (2015). Establishing nurse-led active surveillance for men with localised prostate cancer: development and formative evaluation of a model of care in the ProtecT trial. BMJ Open.

[7] Mcglynn B, White L, Smith K (2014). A service evaluation describing a nurse-led prostate cancer service in NHS, Ayrshire and Arran. Int J Urol Nurs.

[8] Frew LC, Leung SKW (2015). The Fife hormone service for prostate cancer patients: a cost-effective and patient-centric model. Int J Urol Nurs.

[9] James N, McPhail G (2008). The success of a nurse-led, one-stop suspected prostate cancer clinic. Cancer Nurs Pract.

[10] Martin E, Persaud S, Corr J (2018). Nurse-led active surveillance for prostate cancer is safe, effective and associated with high rates of patient satisfaction—results of an audit in the East of England. Ecancermedicalscience.

[11] Faithfull S, Corner J, Meyer L (2001). Evaluation of nurse-led follow up for patients undergoing pelvic radiotherapy. Br J Cancer.

[12] Jones S, Thomas T, Lavelle E (2016). Nurse-led clinic for men receiving targeted therapies for metastatic hormone-relapsed prostate cancer. Cancer Nurs Pract.

[13] Lewis R, Neal RD, Williams NH (2009). Nurse-led vs. conventional physician-led follow-up for patients with cancer. J Adv Nurs.

[14] Leahy M, Krishnasamy M, Herschtal A (2013). Satisfaction with nurse-led telephone follow up for low to intermediate risk prostate cancer patients treated with radical radiotherapy. A comparative study. Eur J Oncol Nurs.

